# Navigating the Surgical Landscape: A Comprehensive Analysis of Endoscopic vs. Microscopic Transsphenoidal Pituitary Surgery Outcomes

**DOI:** 10.7759/cureus.53633

**Published:** 2024-02-05

**Authors:** Abdullah Ashfaq, Syed Faqeer Hussain Bokhari, Abdur Rehman, Amna B Baluch, Ayesha Begum Mohamed Abdul Raheem, Mazin M Almomani, Faisal F Al-Shaikhly, Mohammed Khaleel I. KH. Almadhoun, Muhammad Kamran, Ahsan Shehzad

**Affiliations:** 1 Surgery, Gujranwala Medical College Teaching Hospital, Gujranwala, PAK; 2 Surgery, King Edward Medical University, Lahore, PAK; 3 Surgery, Mayo Hospital, Lahore, PAK; 4 Internal Medicine, Universidad Autónoma de Guadalajara, Guadalajara, MEX; 5 Surgery, Tbilisi State Medical University, Tbilisi, GEO; 6 Medicine and Surgery, University of Jordan, Amman, JOR; 7 Medicine and Surgery, Mutah University, Karak, JOR; 8 Internal Medicine, Mayo Hospital, Lahore, PAK

**Keywords:** acromegaly, cushing's disease, neurosurgery advancements, outcomes analysis, transsphenoidal, microscopic approach, endoscopic approach, pituitary surgery

## Abstract

Pituitary surgery, a critical intervention for various pituitary disorders, has sparked ongoing debates regarding the preference between endoscopic and microscopic transsphenoidal approaches. This systematic review delves into the outcomes associated with these techniques, taking into account the recent advancements in neurosurgery. The minimally invasive nature of endoscopy, providing improved visualization and reduced morbidity, stands in contrast to the well-established track record of the conventional microscopic method. Examining outcomes for disorders such as Cushing's disease and acromegaly, the review synthesizes evidence from Denmark, Bulgaria, and China. Noteworthy advantages of endoscopy encompass higher resection rates, shorter surgery durations, and fewer complications, endorsing its effectiveness in pituitary surgery. While emphasizing the necessity for prospective trials, the review concludes that endoscopic approaches consistently showcase favorable outcomes, influencing the ongoing discourse on the optimal surgical strategies for pituitary disorders.

## Introduction and background

Pituitary surgery is a critical intervention for various pituitary disorders, and the choice between endoscopic and microscopic transsphenoidal approaches has been a subject of ongoing debate within the medical community. This systematic review aims to explore and analyze the outcomes associated with endoscopic and microscopic transsphenoidal pituitary surgery. As advancements in surgical techniques continue to shape the field of neurosurgery, understanding the comparative effectiveness of these two approaches becomes imperative. The endoscopic approach, characterized by its minimally invasive nature, has gained popularity for pituitary surgery in recent years [1]. Proponents argue that it provides enhanced visualization, improved maneuverability, and reduced patient morbidity. On the other hand, traditional microscopic transsphenoidal surgery has been the conventional method for decades, known for its familiarity among surgeons and established track record [2].

Several studies have investigated the outcomes of these approaches in treating pituitary disorders, including but not limited to Cushing's disease, pituitary adenomas, and other tumors. For instance, a systematic review and meta-analysis by Chen et al. compared endoscopic and microscopic transsphenoidal surgery specifically for Cushing's disease, shedding light on the effectiveness of these approaches in managing this specific condition [3]. Moreover, Møller et al. reported promising results for endoscopic pituitary surgery based on the experiences of experienced microscopic pituitary surgeons, indicating a potential shift towards the adoption of the endoscopic technique [1]. Guo et al. conducted a meta-analysis comparing the effectiveness of microscopic and endoscopic surgery for treating pituitary disorders, contributing valuable insights into the overall efficacy of these approaches [4].

This review aims to contribute to the ongoing discourse on pituitary surgery by providing a comprehensive analysis of the outcomes associated with endoscopic versus microscopic transsphenoidal approaches. By synthesizing the existing evidence, we strive to offer valuable insights that can guide both clinicians and researchers in making informed decisions regarding the optimal surgical approach for pituitary disorders.

## Review

Materials and methods

This systematic review strictly adheres to the established Preferred Reporting Items for Systematic Reviews and Meta-Analyses (PRISMA) guidelines, employing a comprehensive approach to investigate the outcomes of endoscopic versus microscopic transsphenoidal pituitary surgery. The subsequent sections delineate the criteria for study inclusion, the search strategy utilized, and the methodology employed for data synthesis.

Search Strategy

We conducted a meticulous search across prominent electronic databases, including PubMed, Embase, and the Cochrane Library, to identify pertinent articles. Our search strategy comprised a combination of Medical Subject Headings (MeSH) terms and keywords related to pituitary surgery, encompassing both endoscopic and microscopic approaches. Boolean operators (AND, OR) were strategically employed to refine the search and identify studies meeting our predetermined inclusion criteria. The search string used for PubMed was ("Outcomes" OR "Treatment Outcome" OR "Surgical Outcome") AND ("Endoscopic Transsphenoidal Pituitary Surgery" OR "Endoscopic Pituitary Surgery" OR "Endoscopic Hypophysectomy") AND ("Microscopic Transsphenoidal Pituitary Surgery" OR "Microscopic Pituitary Surgery" OR "Microscopic Hypophysectomy" OR "Endoscopy"[Mesh] OR "Endoscopy, Surgical"[Mesh] OR "Transsphenoidal Hypophysectomy"[Mesh] OR "Microsurgery"[Mesh] OR "Microscopic Hypophysectomy"[Mesh]).

Eligibility Criteria

Stringent inclusion criteria were predefined to ensure the selection of high-quality and relevant studies. The included studies focused on investigating the outcomes of endoscopic versus microscopic transsphenoidal pituitary surgery. Only articles published in peer-reviewed journals within the timeframe from the inception of relevant databases until October 2023 were considered. Exclusion criteria encompassed studies on other interventions, those lacking sufficient data on surgical outcomes, and studies solely involving animal cells. Additionally, only studies in the English language with full-text availability were included, and gray literature was not considered eligible.

Data Extraction and Synthesis

Two independent reviewers meticulously screened titles and abstracts to identify potentially eligible studies. Subsequently, full-text articles were retrieved and evaluated for adherence to inclusion criteria. Discrepancies between reviewers were resolved through discussion and consultation with a third reviewer. Relevant data, including study design, patient characteristics, interventions, and surgical outcomes, were systematically extracted using a predefined data extraction form.

Data Analysis

A narrative synthesis approach was employed to summarize findings from included studies due to anticipated heterogeneity in study designs and outcome measures. Key themes and patterns related to the outcomes of endoscopic versus microscopic transsphenoidal pituitary surgery were identified and presented.

Results

Study Selection Process

**Figure 1:**
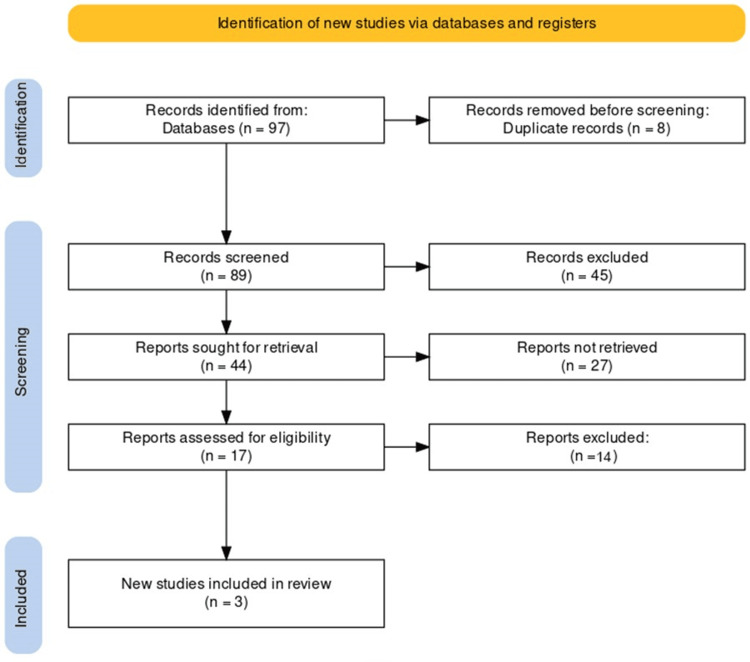
PRISMA flow diagram of the selection of studies for inclusion in the systematic review. PRISMA: Preferred Reporting Items for Systematic Reviews and Meta-Analyses

Following four database searches, 97 articles were initially identified. After eliminating eight duplicates, the titles and abstracts of the remaining 89 publications were evaluated. Subsequently, 17 potential studies underwent eligibility verification through a thorough examination of their full texts. Ultimately, three articles satisfied the inclusion criteria. No additional studies meeting the eligibility criteria were found during the examination of references in the selected articles. The entire process is visually depicted in the PRISMA flowchart (Figure 1).

Characteristics of Selected Studies

Overall, three papers met the inclusion criteria. Two studies were randomized controlled trials (RCTs), one each from Bulgaria and China. One study was an observational study from Denmark. The main findings and characteristics of the included studies are mentioned in the following tables (Table 1 and Table 2).

**Table 1:** Summary of the studies included in this systematic review. RCT: randomized controlled trial; HPA: hypothalamic-pituitary-adrenal; TSH: thyroid-stimulating hormone; HPG: hypothalamic-pituitary-gonadal

Author	Year	Country	Study type	Sample size	No. of participants in the endoscopic group	No. of participants in the microscopic group	Main findings
Møller et al. [1]	2020	Denmark	Observational study	240	45	195	The study comparing endoscopic and microscopic transsphenoidal pituitary surgery revealed that the endoscopic technique exhibited advantages, achieving a higher rate of gross total resection (39% vs. 22%) and shorter surgery duration (86 minutes vs. 106 minutes). Complications within 30 days were lower with the endoscope (17% vs. 27%), and grade II complications or higher were significantly reduced (4% vs. 20%) compared to the microscopic approach. Pituitary function outcomes favored the endoscope, with fewer new deficiencies in the HPA axis (3% vs. 34%) and TSH-dependent deficiencies (15% vs. 38%). The HPG axis also showed better normalization in the endoscopic group (32% vs. 19%). Visual field impairment and postoperative improvement did not significantly differ between the two techniques. Overall, the findings suggest that endoscopic transsphenoidal pituitary surgery may offer superior outcomes compared to the microscopic approach, particularly in terms of resection rates and complication profiles.
Vassilyeva et al. [5]	2023	Bulgaria	RCT	83	43	40	The study compared endoscopic and microscopic transsphenoidal pituitary surgery in acromegaly patients, revealing comparable demographic profiles between the groups. Endoscopic surgery demonstrated advantages with shorter anesthesia and surgery times, as well as a reduced postoperative hospital stay. Complete tumor removal was more frequent with endoscopic adenomectomy, while microscopic surgery showed a higher rate of sub-total removal. Both techniques led to a tendency for somatic improvement, with more pronounced visual function improvement in the endoscopic group. Complications, such as liquorrhea and endocrine disorders, were generally low, with endoscopic surgery showing mainly mild complications. Disease remission rates were similar between the groups at various follow-up intervals. In conclusion, while both techniques proved effective in achieving remission, endoscopic surgery exhibited favorable outcomes in terms of efficiency and some aspects of complication profiles.
Zhang et al. [6]	2021	China	RCT	46	23	23	Endoscopic transsphenoidal pituitary surgery for the treatment of Cushing's disease showed comparable efficacy to microscopic transseptal pituitary surgery but with the added benefits of shorter operative time, reduced estimated blood loss, shorter hospital stays, and fewer complications.

**Table 2:** Summary of demographics, tumor characteristics, and postoperative outcomes of the studies included in this systematic review.

Technique	Møller et al. [1]	Vassilyeva et al. [5]	Zhang et al. [6]
Male-to-female ratio (endoscopic)	25:20	17:26	13:10
Male-to-female ratio (microscopic)	107:88	16:24	12:11
Mean age in years (endoscopic)	61	43.26	55.6
Mean age in years (microscopic)	58	44.12	53.2
Functional tumors (endoscopic)	15	All functional	All functional
Non-functional tumors (endoscopic)	29	-	-
Functional tumors (microscopic)	69	All functional	All functional
Non-functional tumors (microscopic)	115	-	-
Microadenoma size (mm) (endoscopic)	-	4	19
Macroadenoma size (mm) (endoscopic)	-	39	4
Microadenoma size (mm) (microscopic)	-	3	18
Macroadenoma size (mm) (microscopic)	-	37	5
Mean operative time (min) (endoscopic)	86	142	108
Mean operative time (min) (microscopic)	106	176	174
Mean hospital stay (days) (endoscopic)	-	5	2.8
Mean hospital stay (days) (microscopic)	-	7	5.1
Postoperative complications (endoscopic)	2	15	3
Postoperative complications (microscopic)	39	10	8

The quality assessment of the selected studies was done using the Newcastle-Ottawa Quality Assessment Scale. All three studies included in this study turned out to be of high quality with a rating of 9/9 stars (Table 3).

**Table 3:** Quality assessment of the included studies using the Newcastle-Ottawa Quality Assessment Scale.

Author	Selection	Comparability	Outcome	Total stars
Møller et al. [1]	★★★★	★★	★★★	★★★★★★★★★
Vassilyeva et al. [5]	★★★★	★★	★★★	★★★★★★★★★
Zhang et al. [6]	★★★★	★★	★★★	★★★★★★★★★

Discussion

This systematic review thoroughly assesses the effectiveness and results of endoscopic transsphenoidal pituitary surgery in comparison to microscopic transsphenoidal surgery, with a specific focus on pituitary adenomas, including Cushing's disease and acromegaly. The results contribute significant insights into the evolving landscape of pituitary surgery, highlighting the benefits and limitations of both surgical techniques.

The selected studies offer valuable insights into the comparative outcomes. Møller et al.'s observational study in Denmark suggests that endoscopic surgery exhibits superior outcomes with higher gross total resection rates, shorter surgery duration, and fewer complications [1]. Vassilyeva et al.'s RCT in Bulgaria, focusing on acromegaly patients, indicates endoscopic advantages such as shorter anesthesia and surgery times, reduced postoperative stay, and comparable remission rates [5]. Zhang et al.'s RCT in China, specifically for Cushing's disease, suggests comparable efficacy with added benefits favoring endoscopy [6].

The endoscopic approach has been advocated for its panoramic visualization and superior mobility, which are crucial in resecting tumors while preserving normal structures [7,8]. Studies have shown a higher remission rate in endoscopic procedures for endocrine-active tumors, like growth hormone or adrenocorticotropic hormone (ACTH)-secreting adenomas, compared to the microscopic approach [9,10]. Patient comfort and recovery play a significant role in evaluating surgical methods. The endoscopic technique, by avoiding submucosal excision of nasal tissues, typically results in less postoperative pain and rhinological dysfunction. Studies, including ours, have reported shorter operative times and hospital stays with endoscopic surgery, attributed to fewer intraoperative and postoperative complications and a reduced need for wound management [11-13].

Safety is paramount to any surgical intervention. The endoscopic method has shown a decrease in common complications such as cerebrospinal fluid (CSF) leak, pituitary hormone dysfunction, and diabetes insipidus. Additionally, the endoscopic procedure exhibited fewer complications, which could be attributed to the enhanced visualization allowing for more precise tumor excision and preservation of vital structures [14-16].

In the context of acromegaly patients, the endoscopic technique has demonstrated increased radicality in tumor removal. Our review aligns with these findings, showing a higher rate of total tumor resection in endoscopic patients compared to those undergoing microscopic surgery. This improved outcome is likely due to better illumination and a wider angle of vision provided by endoscopic operations [5,17].

The endoscopic technique has shown a statistically significant improvement in visual function post surgery compared to the microscopic method. However, the frequency of certain postoperative complications, such as intraoperative liquorrhea, was higher in microscopic surgery. These differences underline the importance of the surgical technique in influencing the outcomes and complications of pituitary surgery [5,18].

Despite these findings, it is important to recognize the limitations inherent in these studies. Factors such as tumor size, density, and localization significantly influence surgical outcomes and procedure times. Additionally, the retrospective nature of many studies introduces potential biases, underscoring the need for more prospective, randomized trials for a comprehensive understanding of the long-term outcomes of these techniques. 

## Conclusions

This systematic review comparing endoscopic and microscopic transsphenoidal pituitary surgery outcomes indicates consistent evidence favoring the endoscopic approach. Notable studies from Denmark, Bulgaria, and China reveal superior results with endoscopic surgery, demonstrating higher resection rates, shorter surgery duration, and fewer complications. Endoscopy's benefits extend to patient comfort, as evidenced by shorter operative times and hospital stays. Safety considerations also support endoscopy, showing a decrease in common complications such as CSF leaks and hormonal dysfunction. Despite these strengths, the review underscores the need for prospective, randomized trials to address limitations and provide a comprehensive understanding of long-term outcomes.
